# Severe COVID-19 among patients with asthma and COPD: a report from the Swedish National Airway Register

**DOI:** 10.1177/17534666211049738

**Published:** 2021-10-08

**Authors:** Johanna Karlsson Sundbaum, Lowie E.G.W. Vanfleteren, Jon R. Konradsen, Fredrik Nyberg, Ann Ekberg-Jansson, Caroline Stridsman

**Affiliations:** Department of Health, Education and Technology, Luleå University of Technology, Luleå Tekniska universitet, 871 87 Luleå, Sweden; COPD Center, Department of Respiratory Medicine and Allergology, Sahlgrenska University Hospital, Gothenburg, Sweden; Department of Internal Medicine and Clinical Nutrition, Institute of Medicine, Sahlgrenska Academy, Gothenburg University, Gothenburg, Sweden; Department of Women’s and Children’s Health, Karolinska Institutet, Stockholm, Sweden; School of Public Health and Community Medicine, Institute of Medicine, Sahlgrenska Academy, Gothenburg University, Gothenburg, Sweden; Department of Internal Medicine and Clinical Nutrition, Institute of Medicine, Sahlgrenska Academy, Gothenburg University, Gothenburg, Sweden; The OLIN-unit, Division of Medicine, Department of Public Health and Clinical Medicine, Umeå University, Umeå, Sweden

**Keywords:** asthma, COPD, COVID-19, death certificates, hospitalization, obstructive lung diseases, quality register, register studies

## Abstract

**Background:**

Patients with obstructive lung diseases may be at risk of hospitalization and/or death due to COVID-19.

**Aim:**

To estimate the frequency of severe COVID-19, and COVID-19-related mortality in a well-defined large population of patients with asthma and chronic inflammatory lung disease (COPD). Further to assess the frequency of asthma and COPD as registered comorbidities at discharge from hospital, and in death certificates.

**Methods:**

At the start of the pandemic, the Swedish National Airway Register (SNAR) included 271,404 patients with a physician diagnosis of asthma and/or COPD. In September 2020, after the first COVID-19 wave in Sweden, the database was linked with the National Patient Register (NPR), the Swedish Intensive Care Register and the Swedish Cause of Death Register, which all provide data about COVID-19 based on International Classification of Diseases (ICD-10) codes. Severe COVID-19 was defined as hospitalization and/or intensive care or death due to COVID-19.

**Results:**

Among patients in SNAR, 0.5% with asthma, and 1.2% with COPD were identified with severe COVID-19. Among patients  < 18 years with asthma, only 0.02% were severely infected. Of hospitalized adults, 14% with asthma and 29% with COPD died. Further, of patients in SNAR, 56% with asthma and 81% with COPD were also registered in the NPR, while on death certificates the agreement was lower (asthma 24% and COPD 71%).

**Conclusion:**

The frequency of severe COVID-19 in asthma and COPD was relative low. Mortality for those hospitalized was double as high in COPD compared to asthma. Comorbid asthma and COPD were not always identified among patients with severe COVID-19.

## Introduction

At the end of December 2019, the first cases of coronavirus disease 2019 (COVID-19) were reported from China. Since then, COVID-19 has evolved as a major worldwide health crisis.^1^ Although most cases have no or very mild symptoms, the infection can also present as a severe disease with pneumonia, respiratory failure and multi-organ failure, with substantial mortality.^2^ After the first wave, in early September 2020, The National Board of Health and Welfare in Sweden stated that 22,260 patients had been hospitalized with COVID-19 and 5,981 had died.^3^

In addition to older age and male sex,^4^ previous studies have reported an increased risk of severe disease with need for hospitalization and worse clinical outcomes in patients with underlying comorbidities such as hypertension, cardiovascular disease and diabetes mellitus.^5,6^ However, the reported frequency of hospitalized COVID-19 patients with underlying respiratory diseases varies between countries. Studies from both China and Italy have reported low rates (less than 5%) of respiratory diseases among hospitalized patients with COVID-19,^7,8^ while higher figures (14%-18%) have been reported from the United Kingdom and United States.^9,10^ In Sweden, the proportion of patients with chronic lung diseases was 14% in the intensive care units (ICU) during the first months of the pandemic.^11^

Overall, asthma patients do not seem to represent a risk group,^12^ chronic obstructive pulmonary diseases (COPD), on the contrary, has shown to be associated with an increasing need for intensive care treatment and risk of death.^13^ However, most studies are based on patients with COVID-19 identified with asthma or COPD as comorbidities, rather than settings using high-quality nationwide registers for identification of COVID-19 in patients with obstructive lung diseases. The aim of this study was to estimate the frequency of severe COVID-19, and COVID-19-related mortality in a well-defined large population of Swedish patients with asthma and COPD. A secondary aim was to assess the frequency of asthma and COPD as registered comorbidities at discharge from hospital and in death certificates.

## Methods

### Study population – The Swedish National Airway Register (SNAR)

The participants were identified in SNAR, which was initiated in 2013 and includes data on patients with a current physician diagnosis of asthma (children and adults) and/or COPD from primary and secondary care, as well as data on hospitalized COPD patients. In Sweden, the diagnostic criteria for asthma and COPD follow international guidelines.^14,15^ The development and design of SNAR have been described in detail^16^ and the current study was approved by the Swedish Ethical Review Authority (2019-50491; 2020-80050; 2020-70277).

In August 2020, SNAR included in total 271,404 patients, of whom 198,113 (73%) had asthma, 55,942 (21%) had COPD, and 17,349 (6%) had both diagnoses. Patients who had been included in SNAR, but died before January 2020 were excluded from the study population.

### COVID-19 detected by national registers

**Figure 1.**
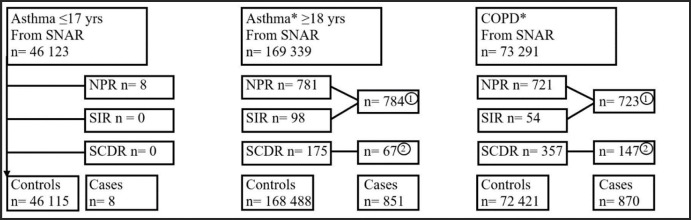
Inclusion of cases and controls.

Data extraction from SNAR was conducted on August 17, 2020, and linked with data from the NPR (inpatient care), the Swedish Intensive Care Registry (SIR), and the Swedish Cause of Death Register (SCDR) on 11 September.^11,17^ These registers provide data about COVID-19 based on International Classification of Diseases, version 10 (ICD-10) codes U07.1 (COVID-19 confirmed by laboratory testing) and U07.2 (COVID-19 clinically or epidemiologically diagnosed, but laboratory testing is inconclusive or not available). The registration of inpatient care, intensive care and causes of death have coverage of nearly 100%.^11,17^ A severe COVID-19 was defined as hospitalization (inpatient care identified as primary discharge diagnosis in NPR and/or ICU registration in SIR) or death due to COVID-19 (registered as an underlying or contributing cause of death in SCDR). Patients in SNAR with severe COVID-19 were defined as cases and patients in SNAR without severe COVID-19 as controls. Figure 1 shows an overview of inclusion of cases and controls from the national registers.

### Asthma and COPD as registered comorbidities in patients with COVID-19

The frequency of asthma and COPD as underlying comorbidities to COVID-19, registered as secondary discharge diagnoses, was retrieved from the NPR at discharge by using ICD-10 codes for asthma J45 (asthma with/ without acute exacerbation) and COPD J44 (COPD with/without acute exacerbation). Report of chronic lung disease among patients receiving intensive care for COVID-19 were analysed from the SIR. From the SCDR, J44 and J45 were used to identify asthma and COPD reported as underlying or contributing causes of death together with COVID-19.

### Statistical analyses

SAS 9.4 for Windows (SAS Institute Inc, Cary, NC, USA) was used for statistical analyses. Frequencies, proportions, means and standard deviations (SD) were used to describe data. For relevant estimates and differences, 95% confidence intervals (CI) were calculated to assess statistical precision.

## Results

### Severe COVID-19 in asthma

Among children with asthma in SNAR, only 0.02% (8 of 46 123 patients) had been hospitalized due to COVID-19. In adults with asthma in SNAR, 0.5% (784 of 168 488 patients) were hospitalized due to COVID-19% and 0.1% (175 of 168 488) died of COVID-19, making the frequency of severe COVID-19% to 0.5% (851 of 168,488) (Figure 1).

**Figure 2.**
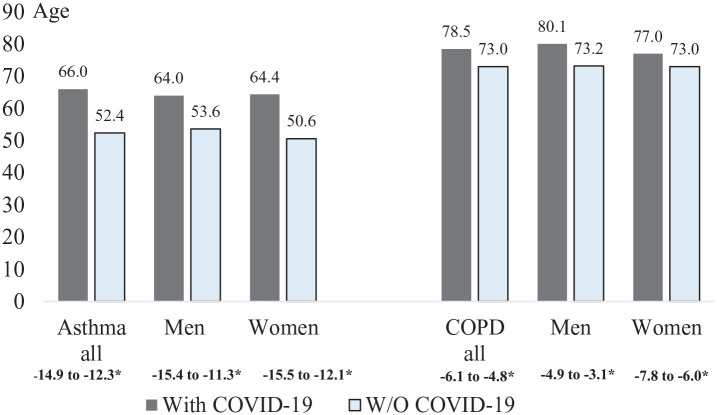
Distribution by age in patients with and without severe COVID-19 infection, in subjects with asthma and COPD, respectively. *Difference 95% confidence intervals (CI).

**Figure 3.**
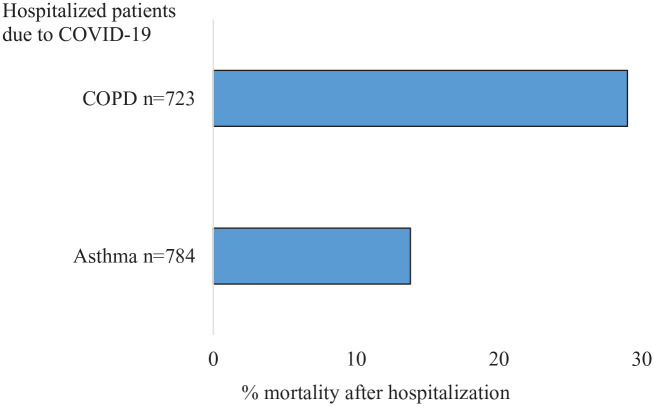
Mortality rate after hospitalization due to COVID-19 in asthma and COPD, respectively.

The proportion of women was equivalent among the cases with severe COVID-19 and controls without severe COVID-19, 59% *versus* 61%, respectively. Regardless of sex, cases with severe COVID-19 were substantially older than controls, with an average age of 66 years, compared with 52 years (Figure 2, Table 1). Among the hospitalized (*n* = 784), 98 patients (13%) received intensive care with an average stay of 13 days at the ICU (Table 2), and 108 (14%) died (Figure 3). No sex-related difference in days at ICU was seen for patients requiring intensive care. The majority of all deaths (in-and out of-hospital) occurred in March-June, and men were on average 5 years younger than women when they died (Table 2). When excluding patients with a diagnosis of both asthma and COPD, the mortality rate among hospitalized asthma patients was slightly reduced to 13%.

**Table 1.** Distribution by sex and age in patients with and without severe COVID-19, in subjects with asthma and COPD, respectively.

Asthma	COVID-19			w/o COVID-19			Difference 95% CI		
	All	Women	Men	All	Women	Men	All	Women	Men
*n* (%)	851	503 (59.1)	348 (40.9)	168,488	102,758 (61.0)	65,730 (39.0)		−1.4 to 5.2	−5.2 to 1.4
Age, *M* (*SD*)	66.0 (17.1)	67.4 (19.5)	64.0 (16.1)	52.4 (19.5)	53.6 (19.4)	50.6 (19.6)	**−14.9 to −12.3**	**−15.5 to −12.1**	**−15.4 to −11.3**
COPD	COVID-19			w/o COVID-19			Difference 95% CI		
	All	Women	Men	All	Women	Men	All	Women	Men
*n* (%)	870	433 (49.8)	437 (50.2)	72,421	41,510 (57.3)	30,911 (42.7)		**4.2 to 10.9**	**−10.9 to −4.2**
Age, *M* (*SD*)	78.5 (9.4)	80.1 (9.1)	77.0 (9.5)	73.0 (9.8)	73.2 (9.8)	73.0 (9.8)	**−6.1 to −4.8**	**−7.8 to −6.0**	**−4.9 to −3.1**

CI, 95% confidence intervals; COPD, chronic obstructive pulmonary diseases.

Bold values indicating significant differences between means or proportions, respectively.

**Table 2.** Distribution of age, sex, care days, death and report of lung diseases in patients with asthma and COPD identified in The Swedish National Airway Register (SNAR) with COVID-19 identified in the National Patient Register, the Swedish Intensive Care Registry, and the Swedish Cause of Death Register, respectively.

Patients from SNAR in the National Patient Register	Asthma with COVID-19				COPD with COVID-19			
	All	Women	Men	Difference 95% CI^a^	All	Women	Men	Difference 95% CI^a^
	789	458 (58.0)	331 (42.0)		721	351 (48.7)	370 (51.3)	
Age, *M* (*SD*)	63.9 (17.6)	65.4 (17.7)	61.8 (17.3)	**−6.1 to −1.1**	77.6 (9.4)	79.1 (9.0)	76.0 (9.5)	**−4.4 to−1.7**
Care days > 18 year, *M* (*SD*)	11.3 (12.9)	10.6 (11.6)	12.0 (14.5)	−0.7 to 3.1	12.9 (12.3)	13.1 (12.2)	12.8 (12.4)	−2.0 to 1.6
Report of ICD J44, *n* (%)	133 (16.9)	73 (15.9)	60 (18.3)	−3.2 to 7.5	586 (81.3)	288 (82.1)	298 (80.5)	−7.2 to 4.2
Report of ICD J45, *n* (%)	445 (56.4)	263 (57.4)	182 (55.0)	−9.5 to 4.6	71 (9.9)	35 (10.0)	36 (9.7)	−4.6 to 4.1
Patients from SNAR in the Swedish Intensive Care Registry	All	Women	Men	Difference 95% CI^a^	All	Women	Men	Difference 95% CI^a^
	98	50 (51.0)	48 (49.0)		54	22 (40.7)	32 (59.3)	
Age, *M* (*SD*)	59.5 (12.7)	59.7 (12.9)	59.4 (12.7)	−5.4 to 4.9	70.9 (7.3)	72.0 (7.0)	70.1 (7.4)	−5.9 to 2.2
Care days, *M* (*SD*)	13.1 (12.3)	12.3 (11.7)	14.0 (12.9)	−3.2 to 6.8	10.4 (12.2)	13.1 (15.5)	8.5 (9.0)	−11.3 to 2.1
Report of chronic lung disease, *n* (%)	70 (71.4)	36 (72.0)	34 (70.8)	−19.1 to 16.8	46 (85.2)	20 (90.9)	26 (81.3)	−27.8 to 8.5
Patients from SNAR in the Swedish Cause of Death Register	All	Women	Men	Difference 95% CI^a^	All	Women	Men	Difference 95% CI^a^
	175	101 (57.7)	74 (42.3)		357	167 (46.8)	190 (53.2)	
Age, *M* (*SD*)	79.5 (11.5)	81.7 (11.0)	76.5 (11.4)	**−8.6 to −1.8**	80.9 (8.2)	82.1 (7.9)	80.0 (8.4)	**−3.8 to −0.4**
Deceased March to April, *n* (%)	85 (48.6)	48 (47.5)	37 (50.0)		174 (48.7)	85 (50.9)	89 (46.8)	
Deceased May to June, *n* (%)	81 (46.3)	47 (46.5)	34 (46.0)		156 (43.7)	67 (40.1)	89 (46.8)	
Deceased July to September, *n* (%)	9 (5.1)	6 (5.9)	3 (4.0)		27 (7.6)	15 (9.0)	12 (6.3)	
Report of ICD J44, *n* (%)	42 (24.0)	26 (25.7)	16 (21.6)	−16.8 to 8.6	252 (70.6)	112 (67.1)	140 (73.7)	−2.9 to 16.1
Report of ICD J45, *n* (%)	42 (24.0)	30 (29.7)	12 (16.2)	**−25.8 to −1.2**	12 (3.4)	6 (3.6)	6 (3.2)	−4.2 to 3.3

CI, 95% confidence intervals; COPD, chronic obstructive pulmonary diseases; ICD, International Classification of Diseases; SNAR, Swedish National Airway Register.

Bold values indicating significant differences between means or proportions.

a Differences between sex, 95% confidence intervals (CI).

### Asthma as registered comorbidity in cases with COVID-19

Among cases with an asthma diagnosis in SNAR, 56% had a record of J45 in the NPR as secondary discharge diagnosis after hospitalization for COVID-19, and 71% of the asthma patients in SNAR attending the ICU were identified having a chronic lung disease in SIR. In cases with asthma diagnosis in SNAR dying from COVID-19, 24% had J45 recorded in SCDR as an underlying or contributing cause of death, with a substantial difference between the sexes (30% women *versus* 16% men) (Table 2).

### Severe COVID-19 in COPD

Among SNAR patients with COPD, 1.0 % (723 of 72 421) were hospitalized, and 0.5% (357 of 72 421) died of COVID-19. The frequency of severe COVID-19 in patients with COPD from SNAR was 1.2 % (870 out of 72 421) (Figure 1). The proportion of men was higher among cases with severe COVID-19 than controls, and the controls were on average 5 years younger than the cases (Figure 2, Table 1). Among the hospitalized (*n* = 721) a total of 54 (7%) cases required intensive care with an average stay at the ICU for 10 days (Table 2), and 210 (29%) died (Figure 3). No sex-related difference in days at ICU was seen for patients requiring intensive care. The vast majority (92%) of all COVID-19 related deaths was reported in March-June, and men were about 2 years younger than women when they died (Table 2).

### COPD as registered comorbidity in cases with COVID-19

In 81% of the cases with COPD diagnosis in SNAR, J44 was recorded in NPR as secondary discharge diagnosis after COVID-19-related hospitalization. Among COPD cases attending the ICU, 85% were identified having a chronic lung disease in SIR. In 71% of the cases, J44 was recorded as an underlying or contributing cause of death in the SCDR (Table 2).

## Discussion

This study describes the frequency of severe COVID-19 in a cohort of over 270 000 Swedish asthma and COPD patients retrieved from the SNAR, a national quality register. Severe COVID-19 was more common in COPD than in asthma, and after hospitalization, 14% of asthma patients and 29% of COPD patients died. Increasing age was associated with severe COVID-19, and men with COPD were more prone to a poor outcome (death). Despite the fact that we identified patients with known diagnosed asthma or COPD who developed severe COVID-19, their underlying comorbid obstructive lung disease was not always registered as a secondary discharge diagnosis or in death certificates, however more often in COPD than asthma.

The proportion of a severe COVID-19 among patients with COPD registered in SNAR seems to be somewhat higher than in the Swedish general population (1.2% COPD in SNAR *versus* 0.3% in the general population ⩾ 18 years),^3^ but with unadjusted data, it is difficult to generalize our results. Today, COPD is well recognized as a comorbidity associated with an increased risk of poor outcome in COVID-19.^15^ As far as asthma is concerned, there is no convincing evidence of asthma as a risk of severe COVID-19,^10,18^ and COVID-19 does not appear to induce severe asthma exacerbations^19^ or increase the risk of worse clinical outcome of COVID-19.^20^ However, our results showed that a higher proportion of patients with asthma required intensive care than patients with COPD, which also is shown in another Swedish study based on ICU patients in relation to COVID-19.^21^ In the latter study, it was speculated that more severe COPD patients could be subject to limitation for care. In our study, patients with COPD were older than those with asthma, and older age may also be a reason for limitation of intensive care. Contrary in US, a higher level of patients seemed to be admitted to ICU due to COVID-19,^22^ which may reflect different management of COVID-19 between countries.^23^

There are studies describing the frequency of lung diseases in severe COVID-19, which are generally low with great variation between studies.^7–10,13,20,24^ It is suggested that under-diagnosis of chronic respiratory diseases, as well as lack of information about comorbidities in medical records, may affect the varying results.^13,24^ Similarly, after severe COVID-19 hospitalization, our result showed a poor agreement between reported hospital discharge diagnoses of asthma and COPD and registered diagnoses in SNAR. However, in the ICU, a higher frequency of chronic lung diseases was reported, suggesting that in this setting both asthma and COPD were generally identified as relevant comorbidities to COVID-19. Previous studies have shown that COPD and asthma are under-reported in death certificates,^25,26^ especially when the primary cause of death is not due to pulmonary diseases.^25^ In our study, the agreement of reported diagnosis of COPD in death certificates and SNAR was 71%, but concerning asthma only 24%.

Our findings are consistent with previous reports that age influences the risk of worse outcome in COVID-19. Children seem to present milder symptoms when infected with COVID-19, and the need for hospitalization is low.^27^ This is in line with our result when only 8 (0.02%) children with asthma in SNAR had been hospitalized due to COVID-19. However, up to date, it is still unknown if asthma is associated with an increased risk of worse outcome in COVID-19 among children.^28^

We have shown that adult patients with severe COVID-19 were significantly older than those without a severe infection, both in asthma and COPD and regardless of sex. At an early stage in the pandemic, reports indicated a poor prognosis in elderly infected with COVID-19^29,30^ and an increased vulnerability in elderly patients suffering from comorbidities such as COPD.^29^ There are reports about elderly patients being at higher risk of pulmonary and cardiac complications^31^ and increased mortality in COVID-19.^32^ This may be the reason for the high mortality rate (nearly 30%) in hospitalized patients with COPD in our study. One explanation to increased risk of poor outcome in the elderly could be the natural aging process of respiratory and immune systems, which might increase the susceptibility to viral infections and lead to more serious clinical outcome.^33–35^

As in other studies,^8,10,36,37^ male sex seems to be associated with severe COVID-19. There are various possible explanations for worse clinical outcomes and higher mortality for COVID-19 infected men than women. The risk of infection after exposure seems to be equal between sexes, but women seem to have better interferon and Toll-like receptor (TLR) mediated anti-viral response and viral clearance. Besides, higher mortality can be associated with increased cytokine concentrations and dysregulated inflammatory response in men, leading to respiratory and cardiovascular complications.^38^

### Strengths and limitations

A major strength is the large database of SNAR with well-characterized physician-diagnosed patients with asthma and COPD. The possibility to link SNAR with other national registers allows monitoring the frequency of a severe COVID-19 infection among asthma and COPD patients in Sweden. Linked together, the current data are a unique resource for respiratory research in patients with obstructive lung diseases. Importantly, the hospital care of patients with COVID-19 varies between countries. For example, the ICU beds are in general fewer in Sweden than in other European countries^39^ and the management of severe COVID-19 is heterogenic^23^ which may affect the external generalization of our results.

As asthma and COPD are chronic diseases with a high prevalence in the population, in Sweden estimated to 10% in asthma and 7% in COPD,^40,41^ a limitation is a difficulty to reach a 100% coverage in SNAR.^16^ However, of all counties in Sweden, health care units in Stockholm and Västra Götaland transmit most frequently data into SNAR, and these counties also have had the highest incident of COVID-19 in Sweden. Thus, over 250 000 patients included from these two counties, together with smaller counties in Sweden, are estimated to be a sample size, large enough, to identify the frequency of a severe COVID-19. Importantly, transmitted data from healthcare systems to the NPR and the National Causes of Death Register are related to a delay. As a consequence of data delay, it may be a risk that our data do not include all cases with severe COVID-19 in Sweden until September. A severe infection may therefore have been under-estimated instead of over-estimated in our study.

Further limitations are no information on the severity of asthma and COPD, and no age-matched control group of non-asthma and non-COPD in this manuscript. Future research will be aimed to study severe COVID-19 in asthma and COPD and the associations with disease severity, pharmacological treatment and comorbidities.

## Conclusion

In Sweden, 0.5% of adults with asthma and 1.2 % of those with COPD in SNAR had a severe COVID-19 during the first wave of the pandemic. Of patients being hospitalized due to COVID-19, 14% of those with asthma and 29% of those with COPD died. Asthma and COPD are not always registered in Swedish health care as comorbidities together with COVID-19, nor as underlying or contributing causes of death. However, COPD is reported more often than asthma, suggesting that physicians in Sweden assess COPD to play a more significant role in the disease course of a severe COVID-19 than asthma.
